# Transformation of Adaptation and Gain Rescaling along the Whisker Sensory Pathway

**DOI:** 10.1371/journal.pone.0082418

**Published:** 2013-12-11

**Authors:** Miguel Maravall, Andrea Alenda, Michael R. Bale, Rasmus S. Petersen

**Affiliations:** 1 Instituto de Neurociencias de Alicante, Consejo Superior de Investigaciones Científicas-Universidad Miguel Hernández, Sant Joan d'Alacant, Alicante, Spain; 2 Faculty of Life Sciences, University of Manchester, Manchester, United Kingdom; University College London, United Kingdom

## Abstract

Neurons in all sensory systems have a remarkable ability to adapt their sensitivity to the statistical structure of the sensory signals to which they are tuned. In the barrel cortex, firing rate adapts to the variance of a whisker stimulus and neuronal sensitivity (gain) adjusts in inverse proportion to the stimulus standard deviation. To determine how adaptation might be transformed across the ascending lemniscal pathway, we measured the responses of single units in the first and last subcortical stages, the trigeminal ganglion (TRG) and ventral posterior medial thalamic nucleus (VPM), to controlled whisker stimulation in urethane-anesthetized rats. We probed adaptation using a filtered white noise stimulus that switched between low- and high-variance epochs. We found that the firing rate of both TRG and VPM neurons adapted to stimulus variance. By fitting the responses of each unit to a Linear-Nonlinear-Poisson model, we tested whether adaptation changed feature selectivity and/or sensitivity. We found that, whereas feature selectivity was unaffected by stimulus variance, units often exhibited a marked change in sensitivity. The extent of these sensitivity changes increased systematically along the pathway from TRG to barrel cortex. However, there was marked variability across units, especially in VPM. In sum, in the whisker system, the adaptation properties of subcortical neurons are surprisingly diverse. The significance of this diversity may be that it contributes to a rich population representation of whisker dynamics.

## Introduction

Adaptation, the accommodation of neuronal responses to ongoing stimulation, occurs across species and sensory modalities [1]. Adaptation implies that the response to any given stimulus depends on the recent history of stimulation. In one prominent form of adaptation which is common across sensory modalities, neurons rescale their firing rate and sensitivity according to the overall scale (variance or contrast) of the ongoing distribution of stimuli [2]–[11]. In the primary somatosensory “barrel” cortex, neurons are tuned to fast temporal features of vibrissa (“whisker”) motion, such as instantaneous velocity [12]–[19]. The gain of this stimulus-response relationship depends on the stimulus statistics of the current sensory environment, such that an increase in the variance of whisker motion causes a compensatory decrease in gain and vice versa [20], [21]. It is not yet known whether these adaptive changes originate in the barrel cortex itself or in the ascending somatosensory pathway. The aim of the present study is to address this issue by analyzing how adaptation to stimulus statistics progresses along the whisker pathway, from the first stage of processing in the trigeminal ganglion (TRG) to the final subcortical relay stage, the ventral posterior medial nucleus of the thalamus (VPM).

## Results

### Response of subcortical units to switching variance of whisker stimulation

**Figure 1:**
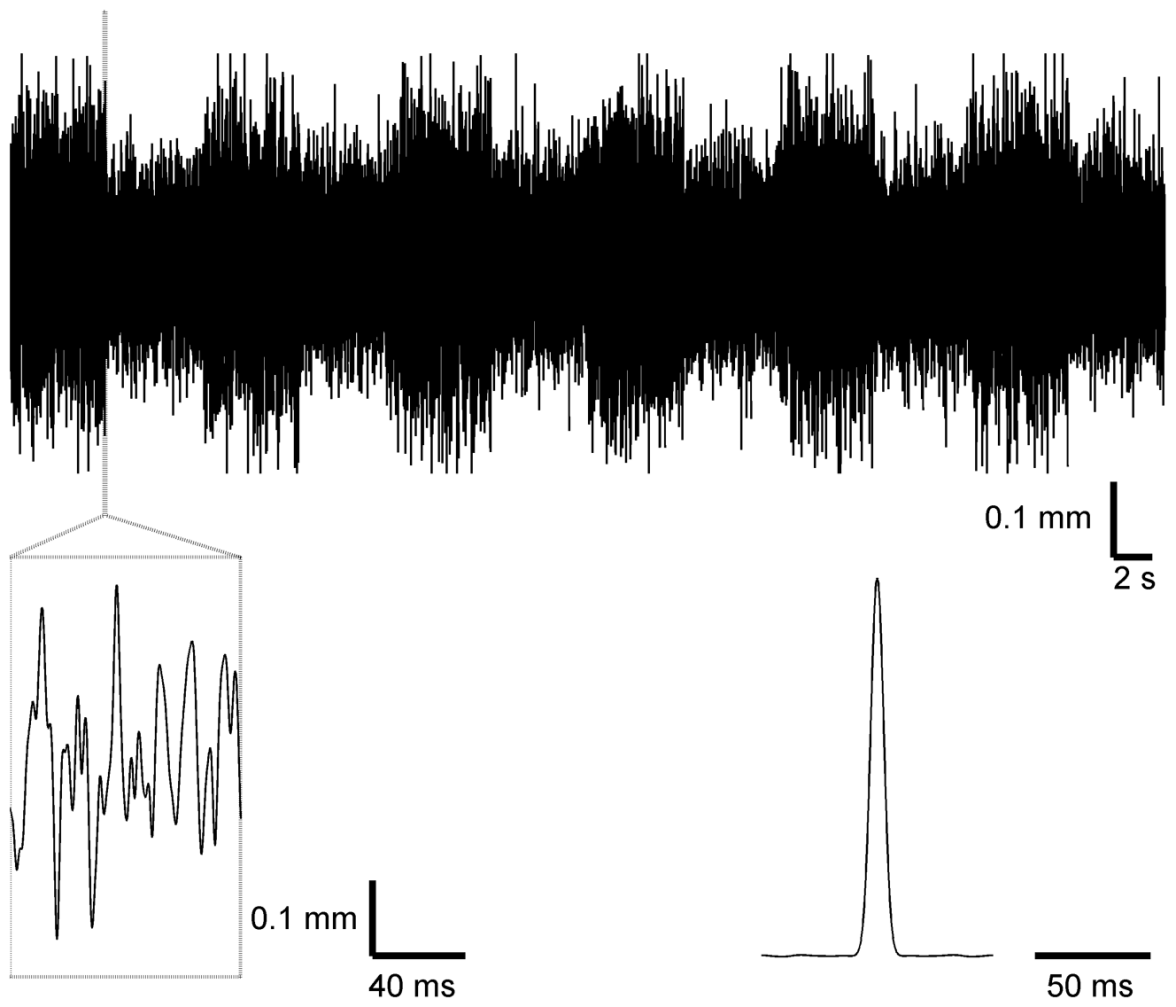
Whisker motion stimulus for probing adaptation. Whiskers were simultaneously deflected with a filtered noise stimulus dorso-ventrally. The variance of the distribution of fluctuations in whisker position switched between ‘low’ and ‘high’ every 5 s. Variance during the high epochs was twice that during the low epochs. Lower left, magnified example of a high- to low-variance transition. Lower right, stimulus autocorrelation function.

**Figure 2:**
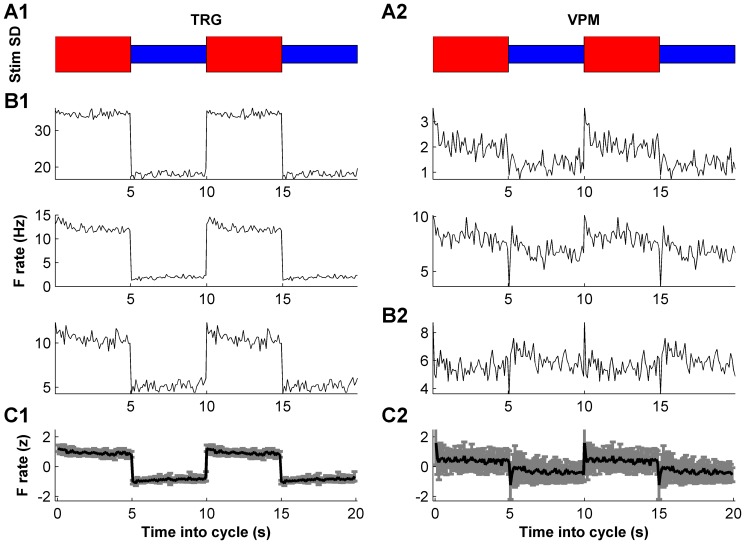
Response of subcortical neurons to variance switching. A1–A2. Schematic of the whisker stimulus showing low and high variance epochs. In order to show both low-to-high and high-to-low transitions, two high-low cycles are shown. B1. Average firing rate evoked by the stimulus for three example TRG units. B2. Corresponding data for three example VPM units. C1. The firing rate of each unit was normalized by converting it to a z-score. Average z-score for TRG. Error bars show SD. C2. Corresponding data for VPM. Note greater variability of rates across VPM units than across TRG.

To determine how adaptation modifies the encoding of whisker motion at different subcortical processing stages in the whisker sensory pathway, we recorded the responses of well-isolated single units from the TRG (n = 11) and VPM (n = 18) of urethane-anesthetized rats (n = 15) to simultaneous deflection of the whiskers with a noise stimulus. Whisker motion consisted of a continuous “noise” trajectory comprising pseudorandom fluctuations occurring on a timescale of a few ms [20], [22], [23]. Additionally, the variance of the distribution of fluctuations changed over a separate, 10 s cycle. Variance switched back and forth between a 5 s “low” epoch and a 5 s “high” epoch, giving rise to two statistical “contexts” within which individual stimulus fluctuations occurred (Fig. 1, Fig. 2A; see Materials and Methods).

Responses both in TRG and VPM were modulated by the high-low variance cycle (Fig. 2B). Firing rates displayed a step-like transition following each switch between low and high variance (Fig. 2B). The step was generally more marked in the TRG units but was clearly observed also in the average VPM response (Fig. 2C).

### Increased diversity in adaptation along the somatosensory pathway

Having established that switches in the variance of the noise stimulus modulated the firing rate of units at multiple stages of the whisker pathway, we tested for adaptive behavior as follows. For each unit, we measured the evolution of the firing rate during the full 10 s period that included the high- and low-variance epochs. In this design, response adaptation would be observed as a systematic change in firing rate within each epoch while variance remained constant (Fig. 2). We found that the amount and temporal evolution of adaptation was variable across units, with clear differences between the populations recorded from the TRG and VPM.

Units in the TRG tended to exhibit comparatively weak adaptation. Typically, their firing rates increased immediately after switching to high-variance stimulation and then plateaued; after stimulation switched back to low variance, firing rates decreased to a lower plateau (Fig. 2B1). Superimposed upon this dominant behavior, a weak, slow decline in response was often observed during the high-variance epoch, and a correspondingly weak recovery was often present during low-variance stimulation. Thus, TRG units exhibited modest firing rate adaptation within each epoch. Although this behavior was quite consistent across units (Fig. 2B1), TRG units did differ substantially in their evoked firing rates. To estimate the average response dynamics, we therefore first converted the firing rate of each unit to a z-score: the resulting curve captured the TRG population's behavior well (Fig. 2C1).

Units in the VPM displayed greater variety of adaptation than TRG units (Fig. 2B2). While units normally reached their peak firing rate soon after the switch from low- to high-variance stimulation, the dynamics governing firing rate decay were diverse. In addition to the slow adaptation components observed in TRG, VPM units often had dynamics featuring both fast and slow structure, sometimes including rebounds in firing rate (Fig. 2B2). In contrast to TRG units, whose firing rate trajectories were well represented by the mean over the population, the trajectories of different VPM units diverged noticeably around the population mean (Fig. 2C2; note larger error bars compared to Fig. 2C1).

**Figure 3:**
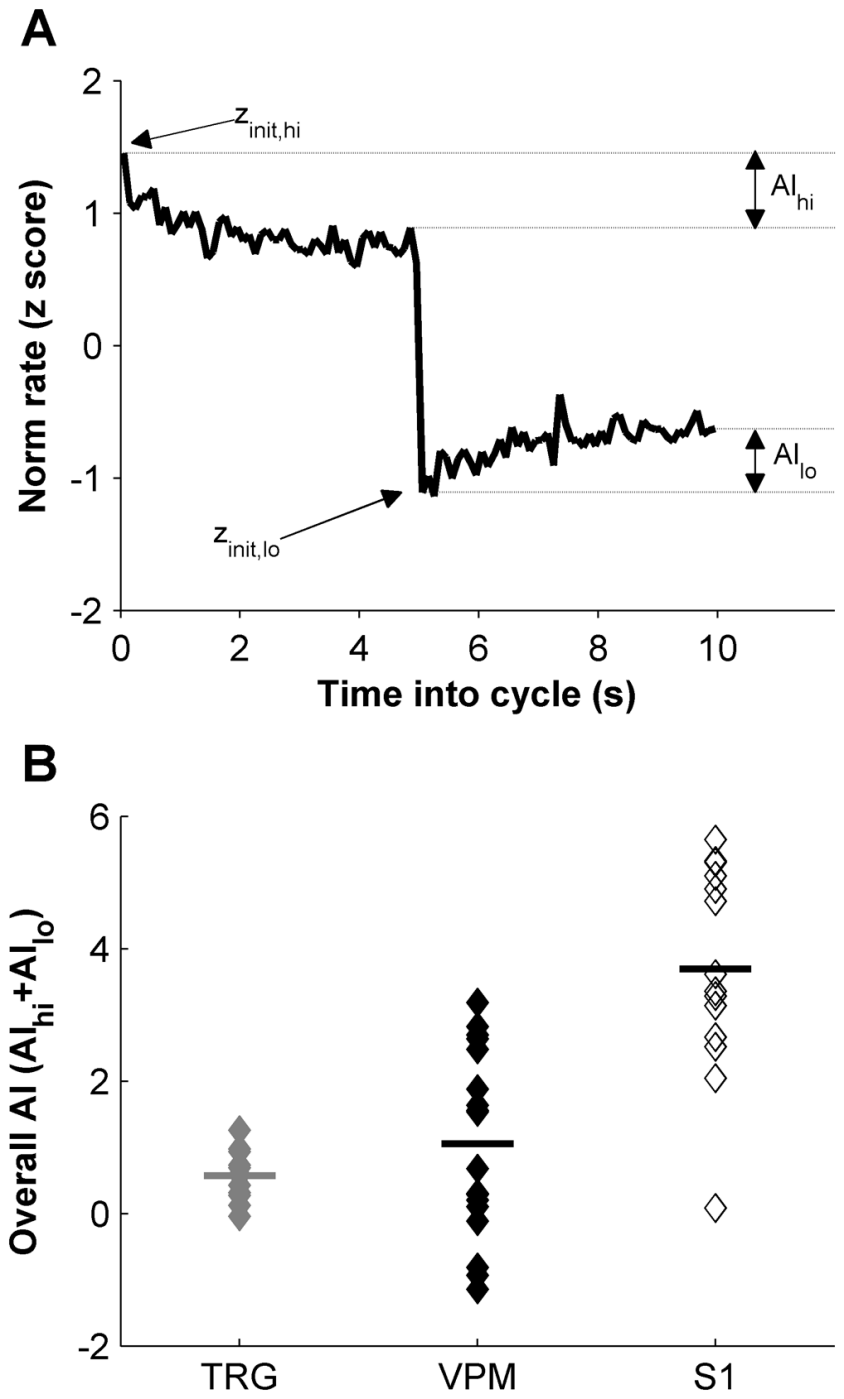
Quantification of firing rate adaptation and comparison across the whisker pathway. A. The firing rate trajectory of an example unit over one high-low variance cycle, normalized as a z-score. Schematic represents computation of adaptation indices (AIs) from z-score rates at different times in cycle (e.g., *z_init,hi_*). *init* signifies rates collected in the first two 100-ms bins within an epoch; *ss* the final five bins within the epoch, corresponding to the steady-state response. B. AI for each TRG unit and each VPM unit in our database. These data are compared to AIs of S1 units previously reported in [20]. Adaptation was more diverse in the VPM than in TRG, but was not significantly higher on average; adaptation did increase on average in S1.

To characterize the diversity of firing rate adaptation at the population level, for each unit we constructed a neuronal adaptation index from the firing rate z-scores. In a typical adapting neuron, the switch to the high-variance epoch elicited a high transient firing rate (*z_init,hi_*) which then decayed to a steady state (*z_ss,hi_*); the switch to the low-variance epoch elicited a low transient firing rate (*z_init,lo_*) which then increased to a steady state (*z_ss,lo_*) (Fig. 3A). We computed a unit's adaptation index for the high-variance epoch as the signed difference between the peak and plateau z-scores (AI*_hi_* = *z_init,hi_* − *z_ss,hi_*, Fig. 3A). The adaptation index for the low-variance epoch was computed analogously (AI*_lo_* in Fig. 3A). Units with large AI*_hi_* also tended to have large AI*_lo_* (n = 29, Pearson r = 0.53, p = 0.0032). Finally, we summed AI*_hi_* and AI*_lo_* to give the overall adaptation index (AI) for the unit, AI = AI*_hi_*+AI*_lo_* = (*z_init,hi_* − *z_ss,hi_*)+(*z_ss,lo_* − *z_init,lo_*). AI values computed in this way gave a robust measure of the amount of rate adaptation displayed by a neuron over the 10 s stimulation cycle. Note that, rearranging terms, AI was equal to the difference in firing rates at the beginning of the high- and low-variance epochs minus the difference in rates at steady state: AI = (*z_init,hi_* − *z_init,lo_*) − (*z_ss,hi_ − z_ss,lo_*). A unit highly responsive to switches between high- and low-variance stimulation and with complete rate adaptation would display a large difference between firing rates at the onset of a switch (i.e., a large *z_init,hi_* − *z_init,lo_*) and no difference between firing rates at steady state (i.e., zero *z_ss,hi_ − z_ss,lo_*), since complete rate adaptation should equalize steady-state responses under different conditions.

Figure 3B shows AI values for all units recorded in TRG and VPM. For comparison, we also plot AI values for a data set recorded in the barrel cortex under equivalent conditions and reported in an earlier publication [20]. Comparing AI values for the TRG and VPM populations revealed greater diversity of adaptation in the VPM (Bartlett's test, p = 0.0003, Fig. 3B), as noted in the examples above. The average level of adaptation was not significantly higher in VPM than in TRG (Kruskal-Wallis with Bonferroni correction for multiple comparisons, p = 0.99, Fig. 3B). In contrast, adaptation increased on average in the barrel cortex compared to both VPM and TRG (Kruskal-Wallis with Bonferroni correction, p = 0.00066 and p = 0.0004 respectively, Fig. 3B). Thus, the change from the TRG to the VPM population was primarily an increase in the variety of dynamic behaviors displayed by different units, while a detectable increase in adaptation level did not occur until the transformation from VPM to barrel cortex.

### Linear-Nonlinear-Poisson framework for describing adaptive changes in stimulus-response relationships

Adaptive alterations in firing rate could imply a change in how a neuron represents a stimulus – for example, a change in the stimulus feature(s) to which the neuron is tuned, or in its sensitivity to those features (reviewed in [1]). To determine which was the case in the present data set, we investigated the stimulus-response relationships of recorded units.

**Figure 4:**
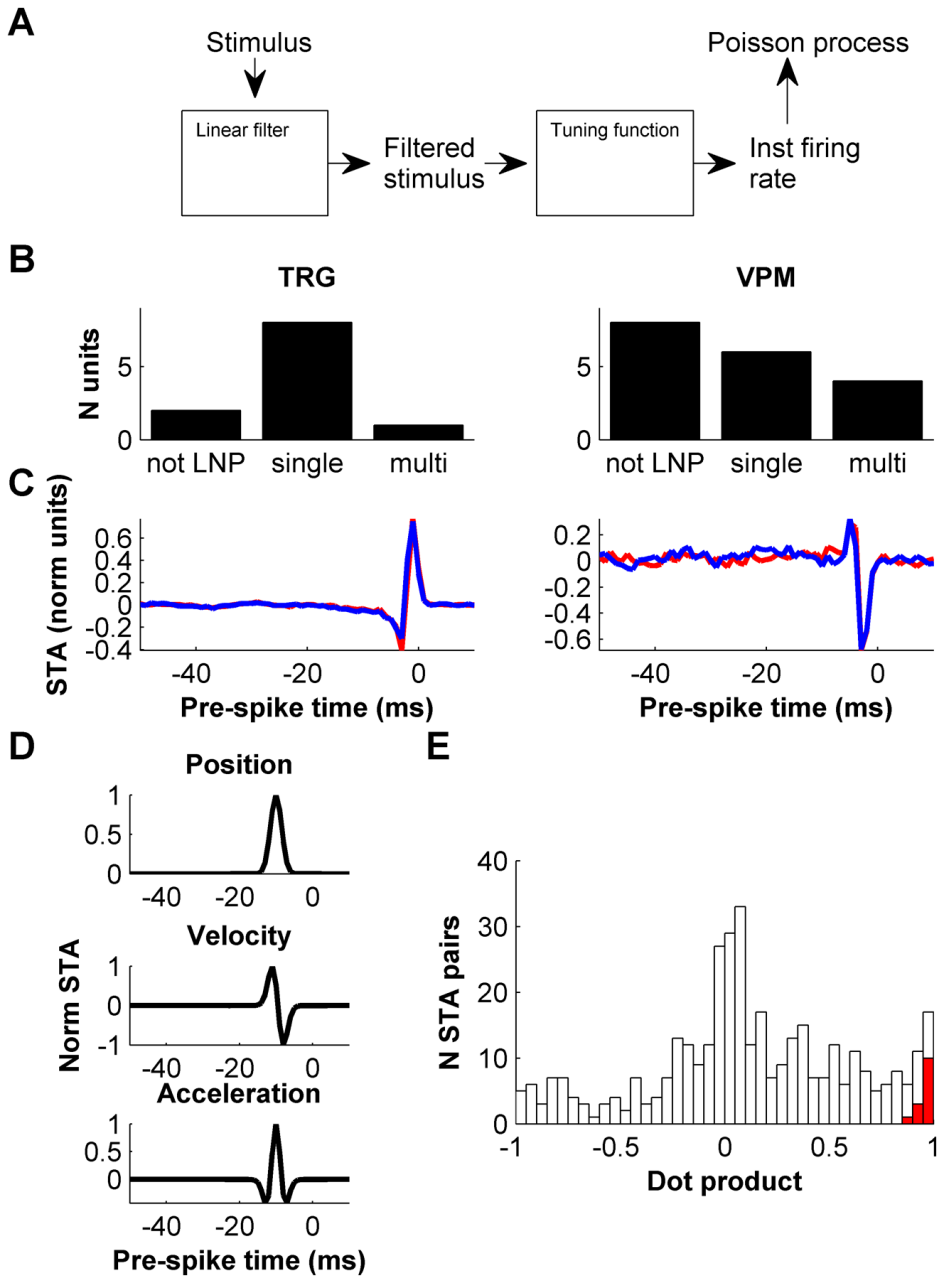
Analysis of adaptation using LNP models: testing for adaptive changes in receptive field. A. Schematic of LNP model. In the L (linear) step, a stimulus time series is convolved with one or more filters. In the N (nonlinear) step, the filtered stimulus is passed through a non-linear tuning function to produce a time-dependent firing rate. Finally, in the P (Poisson) step, this firing rate drives an inhomogeneous Poissonian spike generation process. B. Number of units, in both TRG and VPM that could be well-described (see Materials and Methods) by either an LNP model consisting of a single filter (‘single’), an LNP model consisting of 2 or more filters (‘multi’), or that could not be well-described by any LNP model. C. Filters (STAs) of two example units computed separately using spikes evoked during the high-variance (red) or low-variance (blue) epochs. D. STAs expected from ideal position, velocity and acceleration detectors respectively, given the smoothed white noise stimulus used here [23]. E. Histogram of normalized dot products between the high- and low-variance filters (STAs) of each unit in the TRG and VPM (red), compared to the histogram of normalized dot products between pairs of filters combined at random (white). Dot products between high- and low-variance filters were significantly different than those between pairs combined at random.

Characterizing a neuron's stimulus-response relationship requires (1) identifying the specific stimulus features to which the neuron is selective (its receptive field), and (2) estimating the tuning curve that describes the sensitivity of the neuron's firing rate to the relevant features. Previous work has shown that for both TRG and VPM neurons the stimulus-response relationship can be accurately described by simple, but powerful Linear-Nonlinear-Poisson (LNP) models [23], [24] (Fig. 4A). In the LNP framework, a neuron's feature selectivity is represented by one or more “stimulus filters” [25], [26]. In the simplest (“single-dimensional”) case, which is the most frequent one both in the TRG and the VPM, a neuron's selectivity is well-described by a single filter [23], [24]. The filter(s) are then convolved with the stimulus time series, and firing rate is estimated as a function of the resulting coefficients (see below). Out of the present data set of n = 29 units, n = 19 could be well characterized using the LNP framework (detailed in Materials and Methods; n = 9 out of 11 TRG units; n = 10 out of 18 VPM units; Fig. 4B). In the following, we focus on these well-characterized units.

Both in the TRG and VPM, the majority of units had single-dimensional temporal receptive fields: that is, their feature selectivity was well-described by a single filter recovered by spike-triggered averaging (STA; see Materials and Methods and [23]). Only one ganglion unit out of 9 required a higher-dimensional description involving multiple filters (see Materials and Methods; Fig. 4B). However, a sizable minority of VPM units (4 out of 10) had multi-filter receptive fields (Fig. 4B). This suggests that temporal receptive fields in the whisker system increase gradually in complexity along the pathway, in agreement with earlier findings that essentially all cortical neurons in the system have multi-dimensional receptive fields [19], [20].

To determine whether filters were affected by the state of adaptation, we conducted STA analyses separately for the high- and low-variance stimulation epochs. Examples of filters recovered by STA analysis are shown in Figure 4C. For neurons with single-dimensional receptive fields such as those represented in Figure 4C, the shape of the filter indicates whether the neuron is preferentially selective to stimulus position, velocity, acceleration or other properties. For example, a neuron whose firing rate depended entirely on stimulus velocity (a velocity detector) would have a temporal derivative filter with two equal phases of opposite sign (Fig. 4D middle); in contrast, a neuron acting as a detector of ongoing stimulus position would have a single-phase filter (Fig. 4D top) [23]. We found diverse filters in both the TRG and VPM, in agreement with earlier results [23], [24]. The majority of units had filters with two phases unequal in size (Fig. 4C), indicating that they were intermediate between position and velocity detectors. A minority of units had multiple-phase filters, indicating sensitivity to more complex variables (Fig. 4D bottom). Usually, the duration of the filters (<10 ms; Fig. 4C) was similar to the width of the stimulus autocorrelation (Materials and Methods). This indicates that the timescale of feature selectivity was usually instantaneous to within the resolution of our analysis, in agreement with previous description [23].

### Robustness of feature selectivity to changes in stimulus variance

Adaptation produced no change in feature selectivity. First, units sensitive to a single filter during low-variance stimulation remained sensitive to a single filter during high-variance stimulation. Conversely, every unit that required a multi-filter description (see Materials and Methods) did so under both high- and low-variance stimulation. Second, for single-dimensional units, the filter computed for high-variance stimulation was typically very similar to that computed for low-variance stimulation (Fig. 4C). To quantify this, we computed the similarity (normalized dot product) between the high- and low-variance filters of each unit. The resulting normalized dot product values were always >0.85 and could not be explained under the null hypothesis that high- and low-variance filters were randomly related (Materials and Methods; Kolmogorov-Smirnov, p = 6.0×10^−11^; Fig. 4E). Third, for multi-filter units we computed a measure of similarity between the subspaces spanned by the high- and low-variance filters, known as the subspace projection [27]. The subspace projection is normalized between 0 (no overlap between high- and low-variance subspaces) and 1 (complete overlap), and reduces to the dot product for single-dimensional subspaces. For each multi-filter unit, we computed the subspace projection based on the STA-derived filter plus up to 2 additional filters (Materials and Methods). The resulting overlap between high- and low-variance filter subspaces was 0.85±0.05 (mean ± SEM). We conclude that switches in stimulus variance over the range explored here do not evoke significant adaptive changes in neuronal feature selectivity.

### Changes in stimulus variance induce a rescaling of response gain

Given that stimulus filters were unmodified by changes in stimulus variance, we hypothesized that adaptation to variance could involve changes in tuning curve (second stage of the LNP description; Fig. 4A). In the LNP framework, the probability that a neuron fires an action potential is described by a nonlinear input-output tuning curve that represents the neuron's sensitivity or gain. The tuning curve also captures effects of thresholding, rectification and saturation. Specifically, in the simplest case where the receptive field consists of a single filter, the stimulus time series is convolved with the filter to produce a time-dependent coefficient or filtered stimulus, and the tuning curve predicts firing rate as a function of that coefficient.

**Figure 5:**
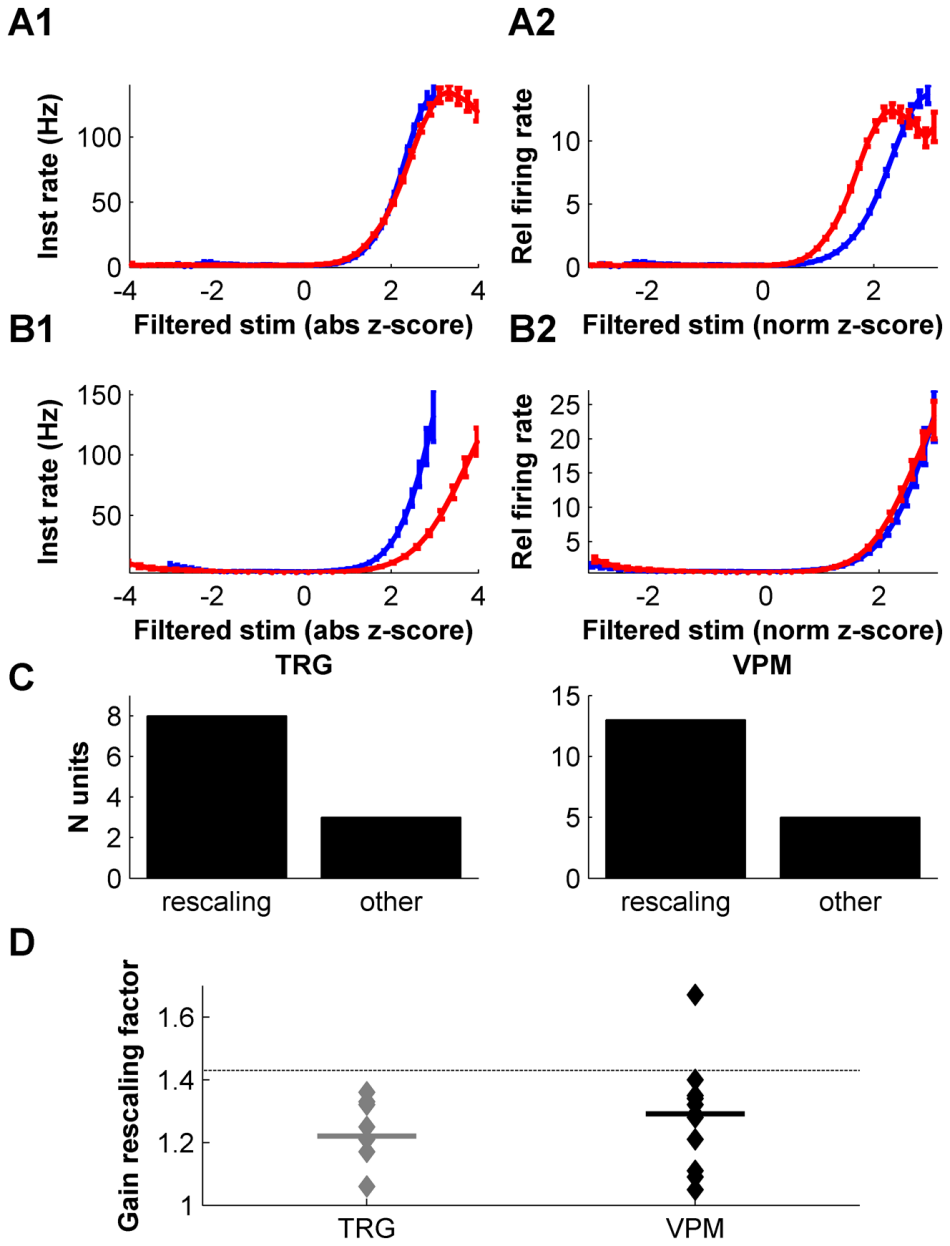
Testing for adaptive changes in neuronal sensitivity (gain). A. Example of a unit whose gain did not adapt. A1. Tuning curve computed from spikes evoked during high-variance epochs (red), compared to tuning curve computed from low-variance epochs (blue). Filtered stimulus is normalized as a z-score. In this plot, the signature of an adapting unit is that the high- and low-variance tuning curves differ. A2. Same tuning curves as A1, but plotted on rescaled axes (detailed in main text). The filtered stimulus (x axis) is normalized by the stimulus standard deviation; the y-scale by the time-averaged firing rate. In this rescaled plot, the signature of a unit that exhibits full gain rescaling (gain proportional to stimulus standard deviation) is that the high- and low-variance tuning curves coincide. B. Corresponding data for an example unit that did adapt. Both this unit and the one in panel A were recorded in VPM. C. Relative number of units for which the residual difference between tuning curves after optimal rescaling was less than 20%, and for which the effect of adaptation was therefore well described as some amount of rescaling (‘rescaling’) compared to those for which the residual difference was greater and for which the effect of adaptation was more complex (‘other’). D. Gain rescaling factor computed as detailed in main text for each unit classified as ‘rescaling’. Factor = 1 corresponds to no rescaling (similar to panel A); factor = 1.43 corresponds to full gain rescaling (similar to panel B).

As with the filter calculation, we determined tuning curves separately for high- and low-variance stimulation epochs (Fig. 5). We plotted the filtered stimulus (x axis) in z-score units, allowing us to express fluctuations in firing rate specifically as a function of stimulus deviations away from the mean. Results for two example units are given in Figure 5A–B. For the unit in Fig. 5A, there was no change in sensitivity: the high- and low-variance tuning curves were identical (Fig. 5A1). In contrast, for the unit in Fig. 5B, tuning curves for the high- and low-variance stimulation epochs differed in scale: the unit was more sensitive during low-variance stimulation, implying an adaptive change (Fig. 5B1). For example, during the low-variance epoch, a firing rate of 80 spikes/s could be evoked by a filtered stimulus value of 2.5 (in z-score units proportional to whisker displacement). To produce the same firing rate during the high-variance epoch, a substantially more intense stimulus was required (filtered stimulus value 3.8). Yet, despite this difference in overall gain, the two tuning curves were remarkably similar in shape (Fig. 5B1). To better visualize this, we rescaled the tuning curves. For the low-variance tuning curve, we normalized the filtered stimulus (x axis) by the stimulus standard deviation of this epoch, and we normalized the firing rate (y axis) by the time-average firing rate in this epoch. The high-variance tuning curve was normalized analogously. In these rescaled coordinates, the tuning curves of the unit of Fig. 5B1 were identical to within measurement error (Fig. 5B2). This suggests that, for this particular unit, the effect of adaptation was simply to rescale neuronal sensitivity to stimulus deviations away from the mean, with no further change in the shape of the nonlinearity [20]. We term this ‘gain rescaling’.

To determine whether gain rescaling was typical across the TRG and VPM populations, for each unit, we tested how well gain rescaling could account for the difference between high- and low-variance tuning curves. To do this, we rescaled one of the tuning curves by a variable factor, searching for the factor that minimized the residual difference (normalized mean squared error) between the rescaled curve and the true tuning curve for the other epoch (Materials and Methods). For most units in the data set, this procedure captured the change in tuning curve remarkably well. Indeed, the resulting residual difference between the true and scaled curves was less than 20% for 21 out of 29 units (73% of TRG units; 72% of VPM units; Fig. 5C). For single-filter units, the residual difference was under 20% for 11 out of 14 units: 7 out of 8 in the TRG and 4 out of 6 in the VPM.

The examples of Figure 5 suggest that subcortical neurons differ substantially in the degree of adaptation that they express. We used the rescaling factor analysis to investigate this. A unit whose tuning curves do not adapt at all corresponds to an optimal rescaling factor of 1; a unit whose tuning curves undergo full rescaling corresponds to a factor equal to the ratio between high and low standard deviations, 1/0.7 = 1.43. For example, the unit in Fig. 5A had very similar tuning curves for low- and high-variance stimulation. Its optimal factor was 1.05, close to 1. In contrast, the unit in Fig. 5B, which exhibited substantial tuning curve rescaling, had an optimal factor of 1.35. In the barrel cortex, the majority of units display full rescaling [20] and this behavior is shared by adaptive neurons in many other systems (reviewed in [1]). Plotting rescaling values for the recorded populations revealed that units in both the TRG and VPM displayed strikingly variable rescaling factors (Fig. 5D). Values outside the range from 1 to 1.43 were possible in principle, but occurred infrequently (Fig. 5D). The distribution of rescaling factors in TRG did not differ significantly from that in VPM (Kolmogorov-Smirnov, p = 0.61). Moreover, interestingly, there was no correlation between the amount of firing rate adaptation and the amount of gain rescaling across units (n = 21, Spearman r = 0.37, p = 0.094). These data indicate that subcortical neurons in the whisker system display adaptive gain control behavior ranging from fixed sensitivity to the absolute stimulus value, to full gain rescaling, with sensitivity normalized by the stimulus standard deviation.

## Discussion

Neurons in the whisker pathway act as fast encoders of dynamic stimulus features such as velocity or acceleration [12]–[19], [23], [28]. Our present findings demonstrate that this feature encoding remains invariant in the face of changes in stimulus scale (variance) throughout the subcortical lemniscal whisker pathway. However, the sensitivity (gain) of neuronal tuning to those features can be altered by changes in stimulus scale. At each observed stage in the pathway (TRG and VPM), different neurons can adjust their sensitivity depending on the scale of the stimulus over a spectrum ranging from no adjustment at all to full adjustment (i.e., the neuron represents the stimulus fully normalized to the current context). Both the first (TRG) and last (VPM) subcortical stages contain neuronal populations with diverse gain rescaling properties, implying that information about both overall stimulus scale and local stimulus fluctuations is preserved and available to downstream neurons. Neurons at these processing stages are also diverse in that they represent different features of a dynamically fluctuating stimulus [23], [24]. In sum, the TRG and VPM each contain a diverse, rich population representation of dynamic whisker stimuli.

### Varieties of adaptation in the whisker pathway

Adaptation in the whisker pathway also occurs under other forms of stimulation (reviewed in [29]). For example, under repetitive stimulation with identical whisker deflections separated in time [12], [30]–[37], neuronal tuning properties are sharpened on successive whisks, including both whisker selectivity [38] and tuning to whisker direction [39]. The ability of neurons to discriminate the relative magnitude of the stimulus improves at the expense of overall stimulus detectability [40]. The extent and time course of adaptation to repetitive stimulation are different at successive stages of the system: more central stages typically undergo stronger adaptation and do so at lower repetition frequencies [33], [35], [38], [41]–[48]. The present study shows subcortical adaptation to changing stimulus statistics and demonstrates an unexpected variety of behaviors across neurons at each stage of processing.

The mechanisms underlying the form of adaptation examined here are unknown. Neurons in the barrel cortex display intrinsic adaptation and gain rescaling to changes in stimulus variance [49], [50], and thalamocortical synaptic depression can also underlie adaptation [33]. It is possible that similar mechanisms act at subcortical stages (e.g., [47]), although we note that adaptation may act through different mechanisms depending on the form of stimulation effectively received by the neuron [29], [49]. We note that adaptive changes in spike rate occurred over a time course long enough to be compatible with possible modulation by corticothalamic feedback.

### Dissection of adaptation with an LNP framework

We used an LNP framework to characterize adaptive changes in neuronal stimulus-response relationships. LNP models have provided a useful way to structure investigations into the nature of adaptation, since they enable its impact to be dissected into effects on a neuron's receptive field (filters) and effects on its tuning curve [7], [8]. Different stimulus protocols affect these two aspects in different ways. In the present study, we found that changes in stimulus variance elicited no modification in receptive field structure, either in receptive field dimensionality or in the shape of filter waveforms. This is common to results in other systems [11], [51], [52]. We also found that switches in variance usually evoked comparatively simple changes in the tuning curves that describe neuronal sensitivity: for most units (21 out of 29), changes in tuning curve consisted of a rescaling of sensitivity or gain (Fig. 5C). For the remaining 8 units, the changes in tuning curve could not be well described as a simple rescaling: the reason was either that the unit was multi-dimensional [23] and a one-dimensional tuning curve analysis could not capture adaptive changes in the underlying multi-dimensional tuning function [52] (4 out of 29), or that the change in shape of the tuning curve could not simply be captured by a linear change of scale (4 out of 29).

Adaptive behavior was diverse across units in our data set. Notably, units varied in the amount of gain rescaling, which covered the entire qualitative range of behaviors from no rescaling at all (rescaling factors ≤1) to full rescaling (factors ≥1.43) (Fig. 5D). While our finding of diverse rescaling is robust to the size of our data set, it is possible that the quantitative range of variation in rescaling factor is wider than reported here.

### Transformations in adaptive behavior across the whisker pathway

We found that the majority of TRG neurons have receptive fields well-captured by a simple, single-filter LNP description (see also [24]); in contrast, a larger fraction of VPM neurons have multi-dimensional receptive fields, whose description required multiple filters. Earlier work found that cortical neurons in the whisker system consistently have multi-dimensional receptive fields [19], [20]. This implies that temporal receptive fields increase in complexity along the whisker pathway, which parallels the behavior of other sensory modalities [53]–[56].

A further interesting comparison can be made between subcortical and cortical tuning curves. Cortical curves typically display rectification and a relatively high threshold, suggesting sensitivity to large excursions in the filtered stimulus [20], [57]. Hence in the barrel cortex, gain rescaling serves to maintain a context-dependent threshold and thus provide sensitivity to relative outliers. In contrast, subcortical tuning curves tend to be more linear in shape, with little rectification and lower thresholds, suggesting faithful representation of filtered stimulus magnitude rather than detection of large-magnitude events [23]. Hence in TRG and VPM, adaptive gain rescaling likely serves to control sensitivity such that stimulus values within the current range are faithfully represented.

Our results show a lower mean amount of firing rate adaptation in the VPM than in the barrel cortex (Fig. 3B). Moreover, barrel cortex neurons display full gain rescaling under the same experimental conditions [20]. This raises the question of whether and how information about absolute stimulus scale is preserved in cortex, which remains an issue for further investigation. Our findings exemplify that the strength of firing rate adaptation need not go hand in hand with the amount of gain rescaling, as there was no significant correlation between the two variables. These two manifestations of adaptation occur conjointly in many systems but may not be mutually required (reviewed in [1]).

## Materials and Methods

### Ethics Statement

All experiments were conducted in strict accordance with international and institutional standards for the care and use of animals in research. Protocols were approved by the UK Home Office and carried out under Project Licence 40/3332. All surgery was performed under urethane anesthesia, and all efforts were made to minimize suffering.

### Experiments

Electrophysiological recordings were made from the TRG and VPM as previously described [58]. Briefly, male adult Wistar rats (*n* = 15; weight 367±22 g SEM, range 245–554 g) were anesthetized with urethane (1.5 g/kg body weight) and placed in a stereotaxic instrument. In any one rat, recordings were made either in the TRG or the VPM. A tungsten microelectrode (8–10 MOhm impedance) was inserted vertically into the brain through a craniotomy using a piezoelectric motor. Extracellular signals were pre-amplified, digitized (sampling frequency 24.4 kHz), band-pass filtered (300–3000 Hz) and continuously stored to hard disk for off-line analysis. Location within VPM was verified electrophysiologically during the experiment and checked by histological identification of the recording site. AC electrolytic lesions were made through the recording electrode by applying 5–10 µA for 15s. After perfusion with 10% formalin, sites were identified by staining 50 µm coronal sections with cresyl violet.

Whiskers were mechanically stimulated as previously described [22]. Briefly, whiskers contralateral to the recorded hemisphere (E1-4, D1-4, C1-4, γ and δ) were cut to 10 mm length and individually placed into the holes of a plexiglass grid, glued to a piezoelectric multilayer bender. The grid was positioned 3 mm from the skin. Motion of the actuator was in the ventro-dorsal direction.

The stimulus was a sequence of pseudorandom white noise with Gaussian amplitude distribution (generated at a sampling frequency of 12.2 kHz), low-pass filtered by convolution with a gaussian kernel (SD 1.6 ms) to restrict stimulus power to frequencies less than the resonant frequency of the mechanical stimulator (300 Hz). Noise was unrepeated, i.e., there were no periods of “frozen” and repeated stimulus trajectories.

The stimulus therefore consisted of fluctuations on a time scale of a few ms. In addition, the amplitude distribution of fluctuations changed cyclically on a separate, longer timescale, switching between a high and a low variance value every 5 s (such that total cycle duration was 10 s, Fig. 1). The low standard deviation equaled 0.7 times the high standard deviation. Each variance switch was smoothed over 10 ms [20]. Both variance conditions had the same frequency spectrum. We verified that the piezoelectric bender accurately reproduced the stimulus by measuring its motion using a custom-built LED-phototransistor circuit [59].

### Analysis

Spikes emitted by individual units were identified by thresholding the extracellular signal and clustering as previously described [22], [23]. VPM units had firing rates ranging from 1.7–17.6 Hz (mean 6.9 Hz, SEM 1.1 Hz). Ganglion units had rates in the range 1.1–40.0 Hz (mean 17.9 Hz, SEM 4.8 Hz).

The evoked spike trains were binned with 100 ms time resolution. The sequence of bins within each stimulus variance cycle was then averaged across cycles to form a firing rate trajectory (Fig. 2). To facilitate comparison of firing rates and of rate adaptation across experiments, we computed each unit's rate as a z-score computed across the stimulus variance cycle. First, we normalized the rate by the total number of spikes in each 10 s cycle and then averaged over cycles to eliminate variations in absolute rate (spike count) over different cycles. Next, we subtracted the rate's mean over the 10 s cycle, and normalized by the standard deviation over the cycle. The resulting z-score gave a specific measure of rate modulation over the course of the cycle. Average population rate plots were prepared by averaging over the z-score plots of units in the population (Fig. 2C).

We characterized neuronal stimulus-response relationships using LNP cascade models as previously described [20], [23] (Fig. 4A). Briefly, for each unit we recovered the spike-triggered stimulus ensemble, i.e., the ensemble of stimuli that evoked spikes, by collecting “snippets” of whisker stimulus waveforms corresponding to the time interval [−50 ms, +10 ms] relative to each spike. Waveforms were binned at 1 ms resolution. Next, we first computed a single filter by spike-triggered averaging (STA) and identified the corresponding tuning curve using Bayes' rule [23]. At this step, units were only kept for LNP analysis if the STA waveform and tuning curve had acceptable (>4x) signal/noise levels, assessed by comparing the magnitude of the STA peak to its variation at baseline (see Fig. 4C for examples). Next, to determine whether the single-filter description sufficed to characterize the unit's feature selectivity, we also computed filters using spike-triggered covariance analysis (STC), which identifies how the distribution of stimuli that elicits spikes differs in shape from the overall distribution of stimuli applied in the experiment [7], [20], [23], [60], [61]. We derived STC filters from the eigenvectors corresponding to significant eigenvalues of the differential covariance matrix, constructed in the stimulus space orthogonal to the STA (as detailed in [23]). If no such eigenvalues were found, we scored the unit as not well described by STC analysis. If significant eigenvalues did exist, the unit's feature selectivity was considered well-described by the STA single filter if the mutual information conveyed about the most significant STC filter was less than 20% of that conveyed about the STA [23]. The subspace projection between high- and low-dimensional sets of filters [27] was computed based on the subspace consisting of the STA-derived filter plus the 2 most significant STC filters, except for 2 units (out of 5) where there was only 1 significant STC filter.

Tuning curves were estimated by application of Bayes' rule [23]. Error bars in Figure 5A,B depict SEM and were constructed by a bootstrap procedure. We determined the extent to which differences between high-variance and low-variance tuning curves could be attributed to gain rescaling, as follows. We multiplied the tuning curve for the low-variance epoch by a rescaling factor and measured the normalized mean squared error (residual) between the resulting rescaled curve and the tuning curve for the high-variance epoch. We then obtained the rescaling factor that minimized this residual.
